# Probing Ligand-Receptor Interaction in Living Cells Using Force Measurements With Optical Tweezers

**DOI:** 10.3389/fbioe.2020.598459

**Published:** 2020-11-17

**Authors:** Carolin Riesenberg, Christian Alejandro Iriarte-Valdez, Annegret Becker, Maria Dienerowitz, Alexander Heisterkamp, Anaclet Ngezahayo, Maria Leilani Torres-Mapa

**Affiliations:** ^1^Institute of Quantum Optics, Leibniz University Hannover, Hannover, Germany; ^2^Lower Saxony Centre for Biomedical Engineering, Implant Research and Development (NIFE), Hannover, Germany; ^3^Institute of Cell Biology and Biophysics, Leibniz University Hannover, Hannover, Germany; ^4^Single-Molecule Microscopy Group, Jena University Hospital, Jena, Germany

**Keywords:** optical tweezers, C-CPE, claudin, force spectroscopy, ligand, cell receptors

## Abstract

This work probes the binding kinetics of COOH-terminus of *Clostridium perfringens enterotoxin* (c-CPE) and claudin expressing MCF-7 cells using force spectroscopy with optical tweezers. c-CPE is of high biomedical interest due to its ability to specifically bind to claudin with high affinity as well as reversibly disrupt tight junctions whilst maintaining cell viability. We observed single-step rupture events between silica particles functionalized with c-CPE and MCF-7 cells. Extensive calibration of the optical tweezers’ trap stiffness and displacement of the particle from trap center extracted a probable bond rupture force of ≈ 18 pN. The probability of rupture events with c-CPE functionalized silica particles increased by 50% compared to unfunctionalized particles. Additionally, rupture events were not observed when probing cells not expressing claudin with c-CPE coated particles. Overall, this work demonstrates that optical tweezers are invaluable tools to probe ligand-receptor interactions and their potential to study dynamic molecular events in drug-binding scenarios.

## Introduction

Dynamic force spectroscopy is a technique which measures the distribution of rupture forces between molecular bonds as a function of loading rate (Capitanio and Pavone, 2013). Along with biomembrane force probe, atomic force microscopy and magnetic tweezers, optical tweezers are one of the few known tools that can be implemented to probe the binding strength of molecular bonds such as cell membrane receptors and ligands (Merkel et al., 1999; Neuman and Nagy, 2008). In optical tweezers, a tightly focused laser beam traps and manipulates microscopic particles with nanometer precision. The optically trapped particle can be additionally functionalized by ligands in order to bind them to receptor proteins present on the membrane of living cells. By monitoring the displacement of the particle from the trap center, the rupture force required to dissociate the bond formed between the ligand and their receptor can be obtained and measured.

A family of membrane proteins, claudins, has been a major interest in cancer community. Claudin proteins are considered the most important component of tight junctions (TJs), structures which form a seal between epithelial cells to limit and regulate paracellular transport (Veshnyakova et al., 2010; Alberts et al., 2002). TJs are essential for the formation of the epithelial barrier in order to prevent uncontrolled flux of substances such as bacterial toxins from the gut lumen into the body (Gunzel and Yu, 2013; Van Itallie and Anderson, 2013). Furthermore, they play a crucial role in the regulation of epithelial cell polarity and the delivery of components to their destination within the plasma membrane (Alberts et al., 2002). TJs are made up of several proteins within both of the two interacting adjacent cells, the majority of which are claudins and occludins (Alberts et al., 2002). Claudins cross the plasma membrane four times with two extracellular loops and C- and N-termini oriented toward the cytoplasm (Mitchell and Koval, 2010). Expression and abundance of claudin subtypes vary depending on the cell and tissue. Since specific claudins such as -3 and -4 are upregulated in some types of cancer cells in prostate, breast, pancreatic and ovary, claudin-targeted therapy is currently under investigation for tumor treatment (Mitchell and Koval, 2010; Gao et al., 2011).

As a potential claudin-targeting ligand, *Clostridium perfringens enterotoxin* (CPE) is a well-known toxin that binds to certain claudins in tight junctions and induces cell death. Understanding of the specific interaction between CPE and receptor claudins could lead to several pharmaceutical applications. A particular interest is the recombinant produced COOH-terminal half of CPE (c-CPE), because it binds to receptor claudins and disrupts tight junctions in a reversible manner, but is not cytotoxic (Gao and McClane, 2011; Shrestha et al., 2016). For example, c-CPE has been shown to sensitize ovarian cancer cells to low dose Taxol, a conventional chemotherapy medication. Fluorescently labeled c-CPE could also be used for tumor imaging (Mitchell and Koval, 2010; Shrestha et al., 2016). Additionally, by inducing TJ disruption, c-CPE enables paracellular transport and thus allows drug delivery beyond physiological barriers (Shrestha et al., 2016). For this reason, it has also been used to transiently open the blood brain barrier and allow diffusion of drugs into non-accessible tissue such as brain parenchyma (Neuhaus et al., 2018). Recently, we have shown that c-CPE functionalized to gold nanoparticles can be used to kill cancer cells upon laser illumination (Becker et al., 2018, 2019).

Although various studies on the interaction of c-CPE to claudins have been conducted in recent years, single molecule binding investigation between this ligand-receptor pair is currently lacking. Insights into the molecular dynamics of the binding event can be obtained using dynamic force spectroscopy methods where increasing force applied to the bond facilitates dissociation (Hinterdorfer and Dufrêne, 2006). Compared to traditional assays, which specify information on dissociation constants based on equilibrium conditions, single molecule studies provide information on the molecular heterogeneity of the complexes formed in real-time, as well as thermodynamic bond parameters such as Gibbs free energy of activation, characteristic length and bond lifetime without force (Stangner et al., 2013).

In this work, we investigate the interaction of claudin and c-CPE by performing force spectroscopy measurements using optical tweezers. In combination with a sensitive back-focal plane interferometric method, we measure the rupture force required to break the claudin and c-CPE bond. Based on the work of Litvinov et al. (2002) and Shergill et al. (2012) we develop a protocol wherein claudin-expressing cells attached to a coverslip are brought into contact with optically trapped particles functionalized with c-CPE and then subsequently retracted. Upon binding, the weak molecular bonds between c-CPE and claudin will exert a force-dependent load on the optically trapped particle until the bond breaks. We describe the calibration process of the trap stiffness and lateral position in order to perform the experiments. Additionally, we detailed the signal processing steps in order to quantify the rupture forces. Overall, this work shows that optical tweezers in combination with back-focal plane detection offer a versatile and precise non-contact method to probe the interaction of ligands with membrane receptors in living cells.

## Materials and Methods

### Experimental Setup

**FIGURE 1:**
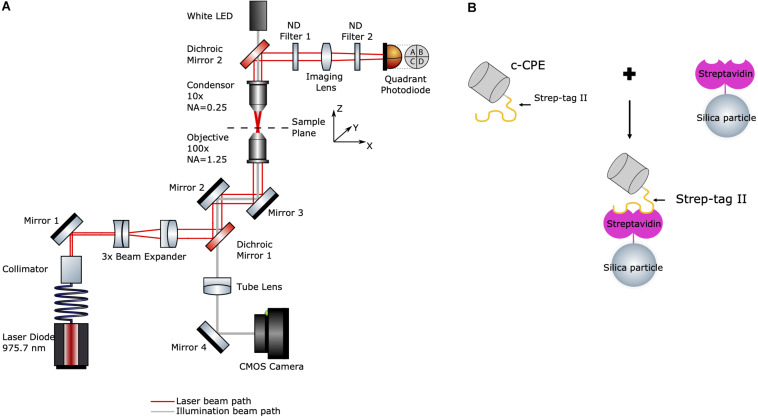
**(A)** Schematic diagram of the optical tweezers setup. Red line indicates the laser beam path starting at the fiber-coupled laser diode (bottom left). Gray line indicates the path of the white LED’s illumination (top). **(B)** Illustration of c-CPE functionalized streptavidin-coated silica particles used in the experiments (drawing not to scale).

The experimental setup is shown in Figure 1A. The optical tweezers system (OTKB/M, Thorlabs, Newton, NJ, United States) with a back-focal plane detection module (OTKBFM) and a force acquisition (OTKBFM-CAL, Thorlabs, Newton, NJ, United States) were used for the experiments. The trapping laser was a laser diode (BL976-SAG300, Thorlabs) controlled with a laser diode/TEC controller (CLD1015, Thorlabs, Newton, NJ, United States) providing a continuous light emission at wavelength 975.7 nm and a maximum output power of 300 mW. The beam was coupled to a single mode optical fiber (SM980-5.8-125) and the output beam was collimated with a triplet collimator (TC06APC-980, Thorlabs, Newton, NJ, United States) and expanded by lenses (*f* = −50 mm and *f* = 150 mm). A shortpass dichroic mirror (DMSP805R, Thorlabs, Newton, NJ, United States) reflected the trapping beam into a high numerical aperture objective (E Plan 100x/1.25 Oil, Nikon) which focused the trapping beam into the sample. A condenser objective (Nikon, E Plan 10x/0.25) collected the light from the sample and along with a biconvex lens (*f* = 40 mm) projects the condenser’s back focal plane onto the quadrant photodiode (PDQ80A, Thorlabs, Newton, NJ, United States) connected to a quadrant detector reader (TPA101, Thorlabs, Newton, NJ, United States). Two neutral density filters (ND) with OD = 0.6 and OD = 0.1 were used to avoid detector saturation. A data acquisition card (USB6212, National Instruments) converted the analog voltage signals to digital values.

For imaging, a white light LED and the condenser objective were used to illuminate the sample. The trapping objective was also used simultaneously to image the sample. An achromatic doublet lens (*f* = 200 mm) acted as a tube lens which projected the image onto a CCD camera (Thorlabs, Thorlabs, Newton, NJ, United States). The trapping events were monitored and recorded with the camera software (ThorCam, Thorlabs, Newton, NJ, United States). Power measurements at the sample plane were performed by a power meter (S121C, Standard Photodiode Power Sensor, Thorlabs, Newton, NJ, United States) by measuring the laser before it enters the objective. After subtraction of the losses due to the objective (according to the manufacturer, 25.5% at 975 nm), a maximum optical power of 168.28 ± 2.49 mW at 450 mA and a lasing threshold current of approximately 45 mA were determined.

The sample was placed on a piezo-driven 3-axis sample stage (MAX311D/M, Thorlabs, Newton, NJ, United States) controlled by 3 piezo controller cubes (KPZ101, Thorlabs, Newton, NJ, United States). Position feedback from two strain gauge readers (TSG001, Thorlabs, Newton, NJ, United States) enabled a step size of 5 nm for lateral movements.

### Back Focal Plane Interferometry

Back focal plane interferometry was used as the position detection scheme. The quadrant photodiode (QPD) in the setup was placed in a plane that is conjugated to the back focal plane of the condenser, where it detected a light pattern caused by interference between light scattered by the trapped particle and the unscattered light (Pralle et al., 1999; Neuman and Block, 2004). The signals X_*vnorm*_, Y_*vnorm*_, and Z_*v*_, which correspond to displacements in lateral X- and Y-direction and in the axial Z-direction, respectively, can be calculated from the raw QPD signals.

The displacements X_*m*_, Y_*m*_, and Z_*m*_, can be acquired by applying conversion factors to X_*vnorm*_, Y_*vnorm*_, and Z_*v*_ (Neuman and Block, 2004; Nicholas et al., 2014). Both position calibration and power spectral density method can be carried out to determine these conversion factors. Since X_*vnorm*_ and Y_*vnorm*_ were normalized by total voltage, their values are given in arbitrary units (A.U.).

### Cell Culture

The epithelial-like human breast adenocarcinoma cell line Michigan Cancer Foundation-7 (MCF- 7, DSMZ no. ACC 115), known to express claudin-3, -4, and -7 (Kominsky et al., 2003; Todd et al., 2015) was used as claudin positive cells. For controls, we used a breast cancer cell line, MDA-MB-231 with minimal expression of claudin -3, -4, and -7 (Becker et al., 2018). Between experiments, the cells were kept in an incubator at 37°C with 5% CO_2_. Cells were cultured in DMEM/F12 (F4815, Biochrom) supplemented with 10% fetal calf serum (S0615, Biochrom) and 1% penicillin/streptomycin (P06-07050, PAN-Biotech). The confluent culture was split every 3 to 6 days. Cells were detached from the culture plate with trypsin/EDTA (PAN-Biotech) for 3 min at 37°C. Trypsin was subsequently deactivated in the solution by adding a double amount of cell culture medium. An aliquot was then transferred into a new tissue culture dish with fresh culture medium. The remaining cells could be used for experiments.

### Media Properties

The characteristics of the cell culture medium in which particles are suspended influences the performance of the optical trap. In particular, its density and viscosity need to be determined to enable the calibration. We measured the dynamic viscosity, η_*cm*_ and the density, ρ_*cm*_ of the medium specified in section “Cell Culture.” The dynamic viscosity of the cell culture medium was measured with a Rheometer Fluids Spectrometer (RFSII, TA Instruments) at room temperature. Using the software RSI Orchestrator, the shear stress was measured for different shear rates. The dynamic viscosity is the slope of the linear fit given by η_*cm*_ = (1.050 ± 0.194) mPa s. Meanwhile the density of the cell culture medium was determined by weighing 1 ml cell culture medium with an analytical balance (M-Pact AX244, Sartorius). Repeating the measurements ten times yielded an average density of ρ_*cm*_ = (1.011 ± 0.006) g/cm^3^.

### Preparation of the *C. perfringens* Enterotoxin C-Terminal Fragment (c-CPE)

c-CPE was prepared as previously described (Becker et al., 2018). In brief, from the genomic *C. perfringens* DNA the c-CPE194-319 gene fragment was PCR amplified and cloned into the pet22b expression vector allowing an N-terminal fusion to the Strep II-Tag. *E. coli* Rosetta pLysSRARE2 transformants expressing the c-CPE Strep Tag-II fusion protein were lysed and the fusion protein was purified using the Strep-Tactin XT Superflow column system (IBA, Göttingen, Germany). After validation, c-CPE was applied at indicated concentration for following experiments. Based on the previously published data (Becker et al., 2018), 5 μg/ml c-CPE was conjugated to Strep-Tactin Chromeo 488 to visualize its binding to MCF7 and MDA-MB-231 cells adhered on a cover slip 2 h after trypsinization.

### c-CPE Functionalized Streptavidin-Coated Silica Particles

A schematic diagram of the particle functionalization is shown in Figure 1B. Streptavidin-coated silica particles (PSI-1.0 SA, Kisker Biotech) with diameter of 955.2 ± 179.3 nm were further coated with c-CPE, which binds to streptavidin via Strep-tag II. 250 μg/ml c-CPE were mixed with 41.5 μg/ml suspended particles in Dulbecco’s phosphate-buffered saline (P04-36500, PAN-Biotech) solution and incubated at 4°C overnight. The day of the experiments, the particles were centrifuged at 12500 *g* for 10 min to remove the unbound c-CPE supernatant and particles were resuspended in fresh cell culture medium.

### Sample Preparation

20 μl solution containing trypsinized cells were transferred onto a 0.17 mm thick glass cover slip (24 mm × 60 mm). Sample was incubated for 30 min at 37°C with 5% CO_2_ to ensure cell attachment to the cover slip surface. Afterward, two pieces of double-sided tape were used to create a small chamber around the sample. Right before the start of the experiment, 10 μl of the particle suspension in cell culture medium (50 μg/ml) were added to the sample. The chamber was closed with a small cover slip (∅ 18 mm) on top.

### Visualization of Binding via Immunofluorescence

5 μg/ml c-CPE was mixed with 5 μg/ml Strep-Tactin Chromeo 488 fluorescent dye and incubated at 4 °C overnight to facilitate binding of Strep-Tactin to Strep-tag II. The following day, the mixture was added to trypsinized cells and incubated for 2 h on coverslips at 37°C with 5% CO_2_. Subsequently, cells were fixed with 4% formaldehyde and stained with nuclear fluorescent dye, Hoechst. As a control, only Strep-Tactin Chromeo 488 without c-CPE was added to the cells. Fluorescence was detected with a inverted microscope (Nikon Eclipse TE2000-E). Chromeo 488 and Hoechst were visualized using light sources with wavelengths, λ = 488 nm and 400 nm, respectively.

## Results

### Calibration of Optical Tweezers

**FIGURE 2:**
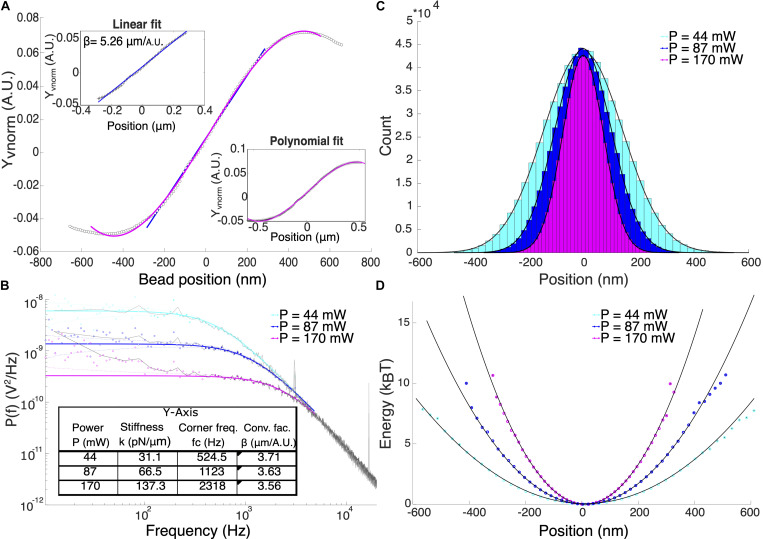
**(A)** Typical position calibration for *Y*-axis of our system. Upper inset shows the linear fit and the lower inset shows the polynomial fit. **(B)** Power spectral density analysis of 1 μm optically trapped particles at different laser power. Colored solid lines are the theoretical Lorentzian spectrum within the range of 10–5000 Hz overlayed to the experimental data (black lines). Filled and hollow symbols are the included and excluded data for the Lorentzian fitting, respectively. Using power spectral density method, both β and trap stiffness can be directly obtained. **(C)** Position histogram of the trapped particles at different laser powers. **(D)** The potential energy in units of *k*_*B*_T shows that at *P* = 170 mW, the potential is harmonic for displacement range of ±300 nm.

The rupture force, *F* was calculated by multiplying the trap stiffness, *k* and the particle displacement, *x*_*m*_ according to Hooke’s law, *F* = *kx*_*m*_. Therefore, an accurate calibration to determine the trap stiffness and particle displacement is crucial for force measurements. The displacement of the particle from the center of the optical trap was determined by applying a conversion factor, β to the respective QPD signals. One way to determine β is to attach a particle to a coverslip and record the QPD signals while the particle is swept through the trap center using a piezo-controlled positioning stage. The normalized QPD signals, X_*vnorm*_ and Y_*vnorm*_ were recorded and a linear curve can be fitted to the signal for small displacements from the trap center as shown in Figure 2A. The slope of this linear fit is the detector sensitivity or the inverse of the conversion factor, 1/β. Position calibration was averaged over 5 different particles. By fitting a linear curve to the signal, the average conversion factors β_*x*_ = (3.05 ± 0.37) μm/_*A.U.*_ and β_*y*_ = (5.04 ± 0.48) μm/_*A.U.*_ for *P* = (168 ± 2.49) mW were obtained. This corresponds to a displacement range of ≈ ± 180 nm in the *x*-axis and ≈ ± 306 nm in the *y*-axis. We attributed the differences in conversion factors for the two axes to the asymmetry of the beam shape at the focus. Detectable position range can be expanded by fitting a third-degree polynomial from the signal maximum to the signal minimum (Nicholas et al., 2014). This method enabled displacement measurements of up to ≈±250 nm and ≈±406 nm for *x* and *y*-axis, respectively.

### Power Spectral Density Method

Power spectral density method (PSD) was used to measure the trap stiffness of the optical trap by analyzing the frequency content of a trapped particle’s Brownian motion. In this method, both the trap stiffness, *k* and the signal to displacement conversion factor, β can be determined simultaneously. PSD method is only valid within the linear range of the QPD which limits the maximum displacement that can be measured with the system. A LabVIEW code was used to record the QPD signals for a trapped particle with an acquisition rate of 100 kHz. The Matlab program tweezercalib2.1 (Hansen et al., 2006) was used to fit a Lorentzian curve to the signal’s power spectral density. The one-sided power spectrum of a trapped particle can be described as a Lorentzian function (Berg-Sørensen and Flyvbjerg, 2004),

(1) $$P\,\left(f\right)=\frac{1}{\beta^{2}}\,\frac{k_{B}\,T}{\pi^{2}\,\gamma \,\left(f^{2}+f_{c}^{2}\right)}$$

where *k*_*B*_ is the Boltzmann constant, *T* is the absolute temperature, *f* is frequency and *f*_*c*_ is corner frequency, γ is the particle’s hydrodynamic drag coefficient given by the Stoke’s law:

(2) $$\gamma =6\,\pi \,\eta \,r_{p}$$

where η is the medium’s dynamic viscosity and *r*_*p*_ is the particle’s radius. Therefore, the medium’s viscosity and the particle radius need to be known. The corner frequency, *f*_*c*_ is related to the trap stiffness, *k* using the following equation,

(3) $$k=2\,\pi \,\gamma \,f_{c}$$

Tweezercalib2.1 accounts for the frequency dependence of the hydrodynamic drag, hydrodynamic interaction with the coverslip, aliasing effects as well as crosstalk between channels. The program computes the corner frequency, from which the trap stiffness [Equation (3)] and a second parameter *D*_*fit*_ in units of A.U.^2^/s for both X_*vnorm*_ and Y_*vnorm*_. This is related to the signal to displacement factor, β given by the following equation,

(4) $$\beta =\sqrt{\frac{D}{D_{f\,i\,t}}}=\sqrt{\frac{k_{B}\,T}{\gamma \,D_{f\,i\,t}}}$$

wherein, D=$\frac{k_{B}\,T}{\gamma }$ is the diffusion constant in units of m^2^/s.

Figure 2B shows the comparison of the trapped particle’s power spectral density at powers, *P* = 44, 87, 170 mW which corresponds to corner frequencies (*f*_*c*_) = 524.5, 1123, and 2318 Hz, respectively. Calculated trap stiffnesses are 31.1, 66.5, and 137.5 pN/μm. All measurements were performed in trapped particles suspended in Milli-Q water using the known values of dynamic viscosity, η_*H_2 O*_ = 1.000 mPa s and density ρ_*H_2 O*_ = 0.998 g/cm^3^. Deviations of the signal from the Lorentzian fit at low frequencies could be due to beam pointing stability and mechanical noise in the system (Berg-Sørensen and Flyvbjerg, 2004). High electronic frequency spikes were observed at 3.06, 16.76, and 33.54 kHz (not shown) that were carried over into the calibration data. These spikes only affect the fitting process when the corner frequency and therefore the trap stiffness is very high (*k* > 150 pN/μm).

### Potential Analysis

Although the displacement of the trapped particle is linear only at a small region where the QPD signal is linear with displacement, it has been shown that the linear range of force is larger. In our case, we validate the range in position at which the trap potential is harmonic, therefore, *F* = *kx*_*m*_ is valid. We obtained the position probability density function (Figure 2C) and calculated the potential energy given by the function: *U*(*x*) = −*ln*(ρ(*x*)). Fitting a parabola, *y* = *ax*^2^ + *b* inform us about the range of position where the optical potential is harmonic as shown in Figure 2D. For *P* = (168 ± 2.49) mW, the potential is harmonic up to at least 10 k_*B*_T within the displacement range ±300 nm for *y*-axis of our system.

### Trap Stiffness Measurements

**FIGURE 3:**
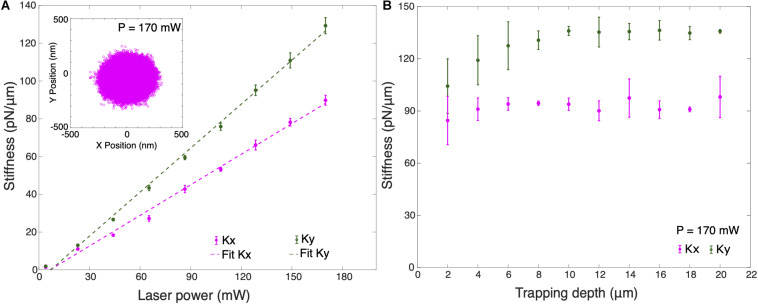
**(A)** Average trap stiffness of 10 optically trapped 1 μm particles for different laser powers. Inset shows the X-Y position plot of the trapped particle at *P* = 170 mW. **(B)** Average trap stiffness as a function of trapped particle height for 5 optically trapped 1 μm particles from the surface of the coverslip at *P* = 170 mW. Error bars are the ±1 standard deviation of the data. All measurements were performed in cell culture medium.

Figure 3A shows the trap stiffness for different optical powers at 10 μm trapping depth. Here, trapping depth is defined as the average height of the trapped particle from the upper surface of the cover slip. Average stiffness values were obtained for 10 different silica particles in cell culture medium using measured values of dynamic viscosity, η_*cm*_ = (1.050 ± 0.194) mPa s and density, ρ_*cm*_ = (1.011 ± 0.006) g/cm^3^, slightly higher than for water. As expected, the trap stiffness has a linear dependence on the optical power. It can be observed that the trap stiffness *k*_*y*_ for the *Y*-axis is larger than the trap stiffness for the *X*-axis by an approximate factor of 1.4. Maximum average lateral trap stiffnesses measured are *k*_*x*_ = (89.81 ± 2.64) pN/μm and *k*_*y*_ = (129.28 ± 3.27) pN/μm in cell culture medium. The trapped particle’s lateral position shows a broader distribution in *X*-axis which confirms the higher trap stiffness in *Y*-axis (inset Figure 3A). This difference in the trap stiffness in X and Y can be attributed to the combined effects of laser polarization (Madadi et al., 2012) as well as to aberrations leading to ellipticity of the focus. We also calibrated the lateral stiffness as a function of trapping depth, averaged for 5 different trapped particles (Figure 3B). The lateral stiffness is approximately constant for trapping depths between 4 and 16 μm. In order to avoid the huge standard deviation in trap stiffness when trapping at shallow trapping depths due to surface effects, all cell experiments were performed at a trapping depth of 10 μm.

### Evidence of c-CPE Binding to Claudin

**FIGURE 4:**
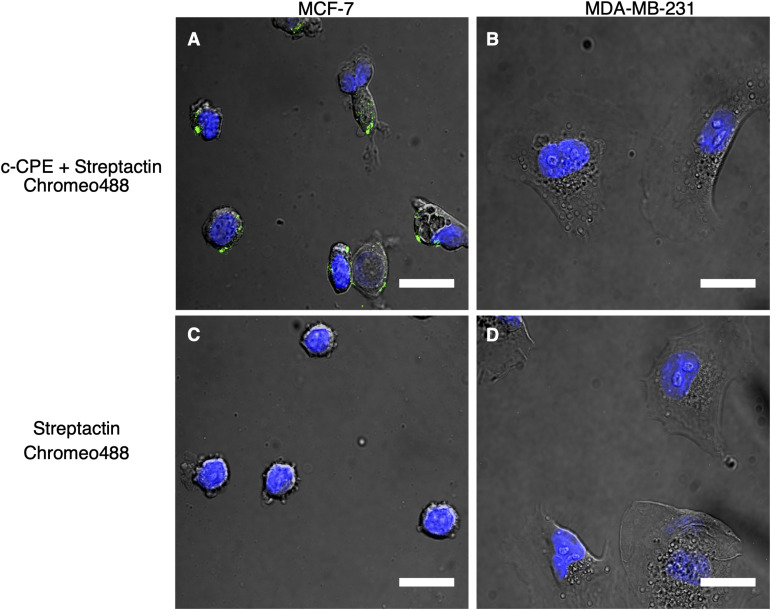
**(A,C)** Specific binding of c-CPE Strep-Tactin Chromeo 488 complex (c-CPE+ StrepTactin Chromeo 488) on claudin-3 and -4 expressing MCF-7 cells (green) 2 h after trypsinization. **(B,D)** No binding of c-CPE on MDA-MB-231 cells were observed. 5 μg/ml c-CPE was conjugated to Strep-Tactin Chromeo 488. Nuclei are stained with Hoechst (blue). Scale bar is 20 μm.

The recombinant protein c-CPE, consisting of c-CPE_194__–__319_ and Strep-tag II was mixed with the fluorescent dye Strep-Tactin Chromeo 488 as indicated in the methods. Figure 4A shows the fluorescence detected in MCF-7 cells. Same treatment was performed on MDA-MB-231 which is shown in Figure 4B. To check for unspecific binding between the Strep-Tactin Chromeo 488 and the cells, both MCF-7 and MDA-MB-231 were also incubated with Strep-Tactin Chromeo 488 without c-CPE as shown in Figures 4C,D, respectively. Green punctate signals indicate the presence of Chromeo 488, and thus c-CPE, whereas the blue corresponds to the cell nuclei. As green dots are clearly visible in the membrane region of the MCF-7 cells but not of MDA-MB-231 cells, as well as in control samples treated with Strep-Tactin Chromeo 488 only, it can be concluded that c-CPE binds specifically to claudin in the membrane of MCF-7 cells.

### Calculation of Force

In order to calculate the total lateral force, a Matlab code was used to convert the recorded voltage signals from QPD, X_*v*_ Y_*v*_, to displacements. The conversion factors in both axes, β_*x,y*_ were obtained from the PSD measurements and V_*sum*_ is the total voltage from the QPD. The displacements, X_*m*_ and Y_*m*_ were calculated as follows:

(5) $$\left[\begin{array}{c} X_{m}\\ Y_{m} \end{array}\right]=\left[\begin{array}{c} \beta_{x}\cdot X_{v\,n\,o\,r\,m}\\ \beta_{y}\cdot Y_{v\,n\,o\,r\,m} \end{array}\right]=\left[\begin{array}{c} \beta_{x}\,\frac{X_{v}}{V_{s\,u\,m}}\\ \beta_{y}\,\frac{Y_{v}}{V_{s\,u\,m}} \end{array}\right]$$

where *X*_*vnorm*_ and *Y*_*vnorm*_ are the normalized voltage signals. The forces F_*x*_ and F_*y*_, in X and Y direction are given by the expression,

(6) $$\left[\begin{array}{c} F_{x}\\ F_{y} \end{array}\right]=\left[\begin{array}{c} k_{x}\cdot X_{m}\\ k_{y}\cdot Y_{m} \end{array}\right]$$

where *k*_*x*_ and *k*_*y*_ are the trap stiffness in X and Y direction, respectively.

As the cell surface is not necessarily perpendicular to the move direction, the total lateral force, $\vec{F_{l\,a\,t}}$ takes into account the contribution of forces from both axes. Finally, the magnitude of the lateral force can be obtained using the following relation,

(7) $$\left|\vec{F_{l\,a\,t}}\right|=\sqrt{F_{x}^{2}+F_{y}^{2}}$$

Therefore, the accuracy and precision of the total force depend largely on the displacement and trap stiffness calibration.

### Measurement of Ligand Binding Strength

**FIGURE 5:**
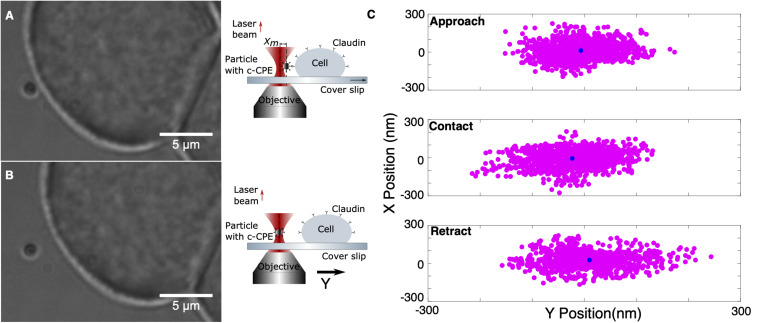
Experimental protocol of ligand binding strength measurement. **(A)** A 1 μm silica particle coated with c-CPE is held in the optical trap. The sample stage is moved to bring an MCF-7 cell into contact with the particle. **(B)** After 1.5 s, the cell is retracted. **(C)** Representative lateral position distribution of the trapped particle during approach (far from the cell), upon contact and immediately after retraction. The black circle shows the mean of the position distribution.

The previously characterized system was used to investigate the binding of c-CPE with claudins. Rupture force experiments consist of two parts, calibration and oscillation. An MCF-7 cell firmly attached to the coverslip was first located and a nearby freely floating particle was trapped at 10 μm trapping depth. The optically trapped particle was then calibrated using sampling rate 100 kHz and sampling time of 6 s. The signal’s power spectral density is automatically displayed after calibration to check for presence of debris or mechanical noise in the system. If the calibration was successful, then force measurements were started. The sample stage and therefore the cell was positioned close to the trapped particle by monitoring the external force on the particle as it was automatically step-wised positioned toward the cell. Upon reaching a force of 5 pN, an automatic oscillation procedure was implemented. The cell stays in contact with the trapped particle for 1.5 s and then retracted with an oscillation amplitude of 5 μm. Since the piezo-driven stage can only be moved in minimum step size of 5 nm, its speed is controlled indirectly via the step size and the time between these steps, 5 ms. All measurements were performed under fixed speed of 1 nm/ms. The movement toward the particle and retraction was repeated for a set number of oscillations, usually 10 for each contact position or until the sequence is terminated manually. Figures 5A,B show exemplary images of the trapping and the corresponding schematic diagram of claudin and c-CPE interaction. When binding between c-CPE and claudin receptor has occurred, upon retraction, the trapped particle is displaced from its equilibrium position. The particle snaps back to the optical trap when the bond dissociates. Figure 5C shows the lateral position distribution of the trapped particle during approach, upon contact and immediately after retraction, including the rupture event. During contact, the position distribution is displaced from the trap center due to the force of the cell membrane on the particle. Directly after retraction, the distribution shifts toward the opposite direction.

**FIGURE 6:**
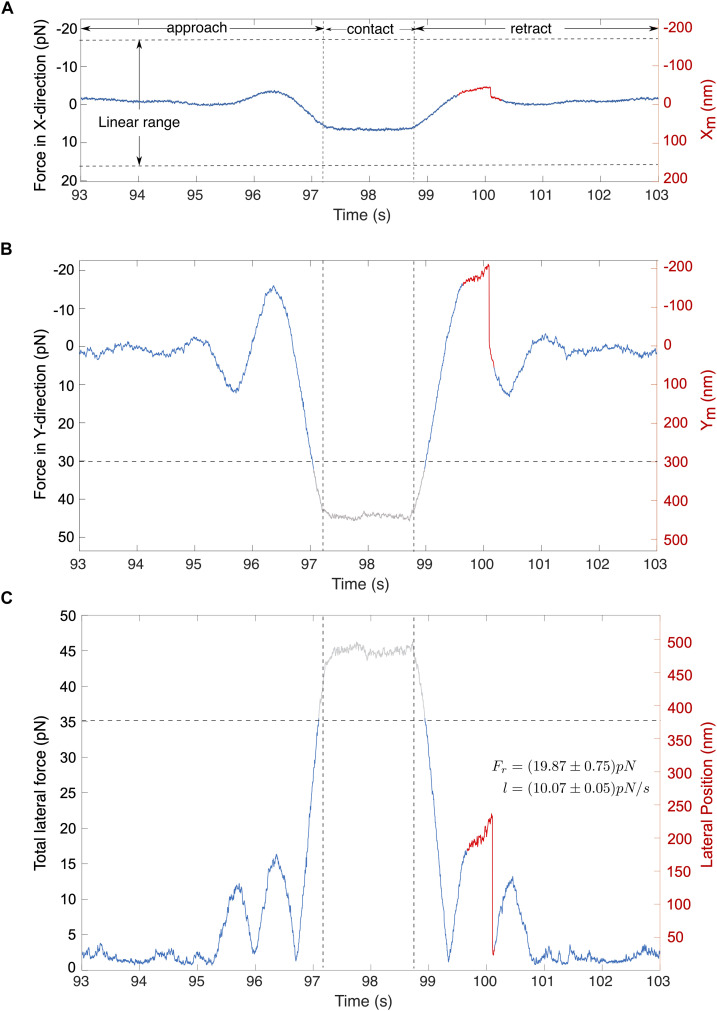
Typical signals for cell-particle contact. The stage oscillates in the direction of the stage’s *Y*-axis. **(A,B)** Traces show the corresponding forces in the direction of X and Y direction of the stage. **(C)** The total lateral force for **(A,B)** is shown, computed using the eq. (7). Abrupt rupture event (marked red) is observed in the total lateral force with a magnitude of *F*_*r*_ = 19.87 pN under a loading force (l) of *l* = 10.07 pN/s. Gray traces represent signals that lie outside the linear range.

A typical force signal is illustrated in Figures 6A–C. In this example, the cell was moved along the stage’s *Y*-axis toward the c-CPE coated particle. Figures 6A,B shows that shortly before the cell contact, the particle experiences an attractive force toward the cell, depicted by a negative force value. As it approaches the cell, this changes into a strong repulsive force. During particle-cell contact, force on the particle becomes constant. While the cell is being retracted, the force reverts into an attractive force resulting in an overall force profile that appears symmetrical. As the cell surface was not perfectly perpendicular to the *Y*-axis, the particle was also slightly displaced in the X-direction (Figure 6A). Absolute values for the total lateral force shown in Figure 6C were computed using Equation (7). Noise was suppressed with a 100th degree median filter.

**FIGURE 7:**
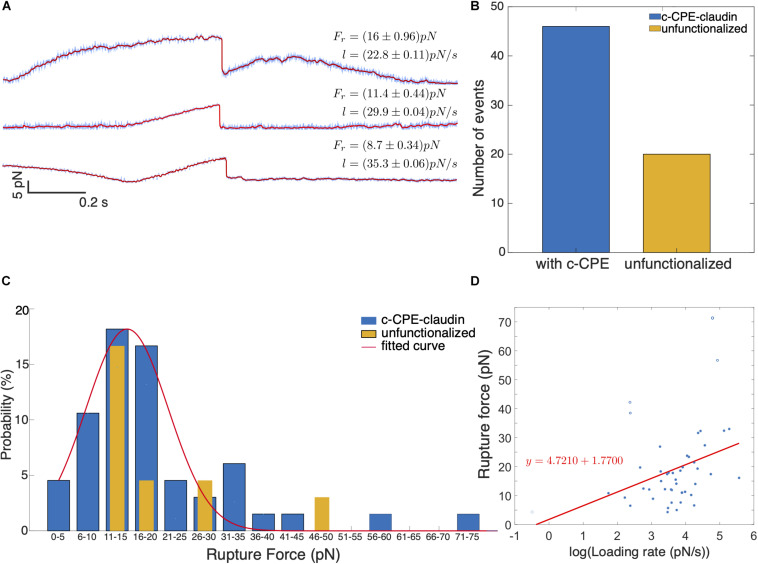
**(A)** Representative time traces of rupture force for varying loading rates. In all binding events observed, a single rupture event occurs which indicates single molecule dissociation between c-CPE and claudin. **(B)** Number of binding events observed with c-CPE and unfunctionalized silica particles. **(C)** Force spectra of rupture events as a function of rupture force binned by 5 pN interval. **(D)** Rupture force as a function of the logarithm of loading rate.

Figure 7A shows example of rupture events with force magnitudes, *F*_*r*_ = 8.7, 11.4, and 16 pN. Prior to most rupture events, a linear increase in force occurs and a sudden drop in force indicates the dissociation of the c-CPE from claudin. In some cases, the increase in force is nonlinear as seen for 16 pN rupture event. Figure 7B shows the summary of all the rupture forces observed for c-CPE coated and unfunctionalized silica particles. The percentage of rupture events out of total contacts performed using particles with c-CPE is ≈ 2.3% (46 out of 2000 contacts). Whereas rupture events using unfunctionalized particles were ≈ 1% (19 out of 2000 contacts) performed in 100 cells for each condition. The number of rupture events increased by more than two-fold when optically trapped particle functionalized with c-CPE was used to probe MCF-7 cells. In contrast, no rupture events were observed when c-CPE coated particle was used to probe MDA-MB-231 cells (1000 contacts, 50 cells).

We plotted the probability of rupture events and binned the data by 5 pN interval. Rupture probability is defined as the percentile ratio of the number of rupture forces to the total number of rupture events. Figure 7C shows the distribution of the rupture forces. For c-CPE coated particles, the rupture forces can be fitted with a Gaussian distribution with a maximum at ≈ 18 pN and a much lower peak at 31–35 pN. Although the maximum probability of rupture events occurs for rupture forces 11–20 pN for both c-CPE coated and unfunctionalized silica particles, a higher probability of rupture force (≈ 17%) can be found at 16–20 pN probed with c-CPE coated particles, 3× more than unfunctionalized particles (≈ 4.5%). For each rupture event, the loading rate is determined from the slope of the increasing force just before the rupture event. Figure 7D shows the rupture forces as a function of the loading rate. For the range of forces obtained in our experiment, the rupture force has a linear dependence with the logarithm of the loading rate.

## Discussion

Ligand binding assays play a crucial role in developing new therapeutic molecules. The binding affinity of a ligand to its target, such as a receptor in cell membrane, is an important parameter in drug development. Quantitative measurements of binding using techniques such as surface plasmon resonance, calorimetry or ELISA provide information on the dissociation constant and are based on ensemble averaging (Benoit, 2011). Some of these methods measure the binding affinity of purified receptor proteins with various ligands in solution. However, not all receptor proteins can be purified and are stably soluble in solution, and even if purified receptor proteins can be produced, they are removed from their native environment which potentially affects their functionality (Helenius et al., 2008). Optical tweezers are able to probe accessible membrane proteins in their natural biological environment providing a functional understanding of their interaction to their specific ligand.

As demonstrated in this work, optical tweezers measure rupture forces between membrane receptors and their ligands with forces less than 100 pN. The calibration of trap stiffness and the displacement of the trapped particles from their equilibrium position play a crucial role in the accuracy of the rupture forces measured. Power spectral density method provides a relatively straightforward method to perform the calibration. It does not require information regarding the viscosity as well as omits the necessity of performing position calibration. In our work, we performed calibration for the trap stiffness and β for every trapped particle used in the cell contact experiments. This approach reduces the uncertainty on these parameters which enables more accurate rupture force measurements.

Since most of the rupture forces obtained in our experiments are below 40 pN, optical tweezers are sufficient for probing receptor-ligand binding interaction at slow loading rates <100 pN/s. In our system, the maximum rupture force measured within the linear range of the displacement measurement is ≈ 40 pN using position calibration. Displacement range increases by performing third order polynomial fit to the lateral position calibration, wherein the measurable maximum rupture force is ≈ 60 pN. However, additional errors are present in position calibration due to the necessity of manual focusing of the particle and the broad particle size distribution. Additionally, the particle used for position calibration cannot be used for cell contact experiments. Using β, as well as *k* directly derived from the PSD for every particle minimizes any error due to particle size variability. By direct force calibration method using a position sensitive detector and a high numerical aperture condenser, it is also possible to obtain a ß value which is robust under changes in particle size and refractive index (Farré et al., 2012).

We demonstrate that specific interaction between c-CPE and claudin receptors in MCF-7 cells can be observed by measuring the single molecule binding events using optical tweezers. Despite the thousands of contacts performed, only limited rupture events were observed (≈2.3%), precluding us from obtaining huge number of measurements for statistical purposes. Yet, rupture forces between c-CPE and claudin in MCF-7 cells can be fitted with a Gaussian distribution profile with a mean rupture force of ≈18 pN. Distribution of forces present when c-CPE functionalized silica particles was used to probe claudin in MCF-7 show smaller rupture forces with magnitudes <10 pN as well as higher probability of rupture forces in the range of 16–20 pN not present in non-functionalized silica particles. Both coated and unfunctionalized particles exhibit high probability of rupture forces between 10 and 15 pN which could be interpreted as non-specific binding between the particles and cell membrane. Nonetheless, the force spectra obtained with c-CPE functionalized particles is distinct from non-functionalized particles.

In comparison to other single molecule binding experiments with optical tweezers, rupture forces for c-CPE and claudin were in the same range of values as other receptor-ligand complexes. For example, rupture forces derived for fibronectin-integrin linkages were 13–28 pN at loading rates of 5–100 pN/s (Thoumine et al., 2000). Meanwhile a force spectrum with a most probable rupture force at 19 pN was obtained from cells expressing Notch ligand Delta-like 1 and Notch 1 functionalized optically trapped particles at loading rates of 250 pN/s (Shergill et al., 2012). Hence, for single molecule experiments with optical tweezers, the probable rupture forces for different receptor-ligand complexes occur within the same range of force values.

The rupture force as a function of the logarithm of loading rate depicts the potential energy landscape of the c-CPE to claudin bond. The Bell-Evans model, describes a single barrier potential wherein the rupture force, *F*_*r*_ exponentially increases as a function of the rate of applied force, more commonly known as the loading rate, *l*, given by the equation,

(8) $$F_{r}\,\left(l\right)=\frac{k_{B}\,T}{x_{\beta }}\,l\,n\,\left(\frac{l\,x_{\beta }}{k_{o\,f\,f}^{0}\,k_{B}\,T}\right)$$

where *k*_*B*_ is the Boltzmann’s constant, T is the absolute temperature, *x*_β_ is the distance between bound and transition state (or the potential width) along the reaction coordinate and $k_{o\,f\,f}^{0}$ is the zero-force dissociation constant (Evans and Ritchie, 1997; Merkel et al., 1999). The extrapolated values *x*_β_ and $k_{o\,f\,f}^{0}$ quantify the energy landscape and the kinetic parameters of the bond complex, respectively.

Using linear regression model, we extracted the values *x*_β_ = (0.872 ± 0.34) nm and $k_{o\,f\,f}^{0}$ = (0.1455 ± 0.2) s^–1^ for c-CPE and claudin bond. These values are within the same range derived from dynamic force measurements using optical tweezers measured for other bond complexes (Arya et al., 2005). Dissociation constant at zero-force for c-CPE and claudin 9 measured using bio-layer interferometry method is $k_{o\,f\,f}^{0}$ = 1.67 × 10^– 4^ s^–1^ (Vecchio and Stroud, 2019). In comparison to our extrapolated $k_{o\,f\,f}^{0}$, the value obtained from ensemble- based measurement is three orders of magnitude smaller, implying a higher bond affinity and a much slower dissociation time constant. However, it has been known that dissociation constants at zero force derived among different single molecule studies can vary and can have huge discrepancies to ensemble measurements (Rico et al., 2019). For example, for the well-characterized high affinity streptavidin-biotin bond, the dissociation constants range from $k_{o\,f\,f}^{0}\approx 1$ to 10^−6^ s^– 1^ measured by atomic force microscopy (AFM) (Yuan et al., 2000; Taninaka et al., 2010; Rico et al., 2019). On the other hand, ensemble bond lifetime measurements of off-rate can vary between ≈10^−4^ to 10^−6^ s^– 1^ (Deng et al., 2013). Techniques used for ensemble measurements have their own limitations and must be kept in mind when comparing with single-molecule results especially at extremely low $k_{o\,f\,f}^{0}$ values (Morfill et al., 2007).

The stochastic nature of single molecule binding typically requires significantly large number of rupture events to accurately model the response of the bond complex to force (Hinterdorfer and Dufrêne, 2006; Johnson and Thomas, 2018). In our experiments, this is quite challenging since not every contact induces binding resulting to bond dissociation. The one barrier model, we have used as well as many others, provides a simplistic approach to quantitatively measure the bond energy profile. Yet, reports have also shown that bond rupture may be more complex and that force applied could introduce other events such as intermediate states during unbinding (Rico et al., 2019), formation of catch bonds (Marshall et al., 2003), distortion of energy pathway (Suzuki and Dudko, 2011) or generation of multiple energy barriers (Merkel et al., 1999; Boye et al., 2013). Since the observed rupture forces have a one-step profile, we interpreted these dissociations as single molecule-rupture events. Future single-molecule experiments would be needed in order to fully elucidate the complex interaction between c-CPE and claudin.

## Data Availability Statement

The raw data supporting the conclusions of this article will be made available by the authors, without undue reservation, to any qualified researcher.

## Author Contributions

MT-M, AH, and AN conceived the experiments. MT-M and AN designed the experiments. CR and CI-V performed the experiments and analyzed the data. AB performed immunostaining and produced the c-CPE. MT-M, CR, CI-V, and MD interpreted the results. MT-M and CR drafted the manuscript with contribution from all co-authors. All authors read and approved the manuscript.

## Conflict of Interest

The authors declare that the research was conducted in the absence of any commercial or financial relationships that could be construed as a potential conflict of interest.
